# Targeting lipid biosynthesis pathways for hepatitis B virus cure

**DOI:** 10.1371/journal.pone.0270273

**Published:** 2022-08-04

**Authors:** Anastasia Hyrina, Dara Burdette, Zhijuan Song, Ricardo Ramirez, Ayse Okesli-Armlovich, Archana Vijayakumar, Jamie Bates, James L. Trevaskis, Simon P. Fletcher, William A. Lee, Meghan M. Holdorf

**Affiliations:** Gilead Sciences, Inc., Foster City, California, United States of America; Centre de Recherche en Cancerologie de Lyon, FRANCE

## Abstract

Chronic hepatitis B virus (HBV) infection is characterized by the presence of high circulating levels of non-infectious lipoprotein-like HBV surface antigen (HBsAg) particles thought to contribute to chronic immune dysfunction in patients. Lipid and metabolomic analysis of humanized livers from immunodeficient chimeric mice (uPA/SCID) revealed that HBV infection dysregulates several lipid metabolic pathways. Small molecule inhibitors of lipid biosynthetic pathway enzymes acetyl-CoA carboxylase (ACC), fatty acid synthase, and subtilisin kexin isozyme-1/site-1 protease in HBV-infected HepG2-NTCP cells demonstrated potent and selective reduction of extracellular HBsAg. However, a liver-targeted ACC inhibitor did not show antiviral activity in HBV-infected liver chimeric mice, despite evidence of on-target engagement. Our study suggests that while HBsAg production may be dependent on hepatic de novo lipogenesis *in vitro*, this may be overcome by extrahepatic sources (such as lipolysis or diet) *in vivo*. Thus, a combination of agents targeting more than one lipid metabolic pathway may be necessary to reduce HBsAg levels in patients with chronic HBV infection.

## Introduction

Hepatitis B virus (HBV) is a global public health threat accounting for the majority of viral hepatitis related deaths. The worldwide estimated prevalence of HBV infection is 3.5%, with 257 million people living with the chronic infection [1]. A hallmark feature of chronic Hepatitis B (CHB) is the presence of high circulating levels of non-infectious small lipid HBV surface antigen (HBsAg) vesicles, which is associated with a dysfunctional HBV-specific host immune response [2–5]. Persistence of CHB is attributed to the maintenance of an intrahepatic pool of the viral covalently closed circular DNA (cccDNA), which serves as the transcriptional template for all viral gene products required for replication including HBsAg [6]. In addition to cccDNA, integration of HBV DNA sequences into the host genome also contribute to viral persistence and is a major source of HBsAg production [7]. Nucleos(t)ide analogue (NA) treatment is effective at preventing infectious virus production and spread but has no direct impact on cccDNA levels or the expression of HBsAg and other viral gene products.

During CHB, HBsAg is expressed in large excess and circulates within small, non-infectious lipid vesicles; ~ 25% of the HBsAg molecular weight is composed primarily of phospholipids (60%), cholesterol (15%), cholesteryl esters (14%), and triglycerides (3%) [8]. HBsAg is initially synthesized as a transmembrane protein within the endoplasmic reticulum, and eventually becomes integrally associated with the lipid bilayer leading to the formation of mature lipoproteins [9]. High levels of circulating HBsAg have been shown to lead to chronic immune dysfunction and failure to eliminate the virus. While HBsAg seroclearance is rarely observed in CHB patients on long-term NA therapy, it is associated with decreased rates of cirrhosis and hepatocellular carcinoma [10, 11]. Therefore, sustained HBsAg loss is considered an important clinical endpoint for HBV therapies and a key goal of functional cure [12].

All viruses rely upon host pathways for successful replication, and there is increasing interest in identifying these pathways as novel therapeutic targets for antiviral drug discovery. Recently, a number of new host factors essential for HBsAg production were identified through a genome-wide CRISPR screen including a number of lipid related genes [13]. Interactions between hepatic lipid metabolism and HBV infection has previously been described in the literature [14, 15] and it has been demonstrated in vitro that depletion of cellular cholesterol using lovastatin, a competitive inhibitor of 3-hydroxy-3-methylglutaryl coenzyme A reductase, significantly reduced HBsAg secretion in Hep3B cells [16].

Hepatic lipid homeostasis is mediated by a balance between the rate of fatty acids (FA) in input (uptake and de novo lipogenesis (DNL) with subsequent esterification to triglycerides) and the rate of FA output (oxidation and secretion) [17]. DNL is controlled through a complex cytosolic polymerization in which acetyl-coenzyme A (CoA) is converted to malonyl-CoA by acetyl-CoA carboxylase and undergoes several cycles of metabolic reactions to form one palmitate molecule. DNL is regulated by the FA synthase (FASN) complex, acetyl-CoA carboxylase (ACC) 1 and 2, diacylglycerol acyltransferase (DGAT) 1 and 2, stearoyl-CoA desaturase (SCD) 1, and several nuclear transcription factors (sterol regulatory element binding proteins [SREBPs], carbohydrate responsive element binding protein, liver X receptor α, farnesoid X receptor, and peroxisome proliferator-activated receptors) [18].

To determine if HBV infection modulates host lipid pathways modulated by HBV infection, we performed metabolomic and RNAseq analyses of liver tissue samples from chimeric humanized mice with and without HBV infection. Also, to evaluate if perturbation of lipid biosynthetic pathways could interfere with HBV infection and/or HBsAg secretion, we assessed activity of small molecules targeting key enzymes within lipid biosynthesis. Using these approaches, we observed that HBV infection dysregulated several lipid metabolic pathways, and small molecules targeting key enzymes within DNL, including ACC1/2, potently inhibited HBsAg secretion in *de novo* infected HepG2-NTCP cells. Surprisingly, *in vivo* administration of a well characterized liver-targeted ACC inhibitor with confirmed on-target activity showed no antiviral activity in liver chimeric humanized mice. These results suggest that targeting this pathway for HBsAg production may not be sufficient *in vivo* where lipids might be derived from other sources such as lipolysis or diet and warrants further investigation.

## Materials and methods

### Generation of humanized mice, infection, and drug administration

Human liver-chimeric uPA/SCID mice were generated by PhoenixBio Co., Ltd. (Higashi-Hiroshima, Japan) as previously described [19]. Human hepatocytes were derived from donor BD195 (BD Biosciences, Woburn, MA). The use of these cells for the generation of chimeric humanized mice has been reported previously [20]. Mice containing human hepatocytes with a mean estimated replacement index of 86% (range 83–90%), calculated based on the blood concentration of human albumin [h-Alb], were infected with 1x10^5^ copies/mouse of HBV genotype C and followed for eight weeks to establish persistent infection of the human hepatocytes in the chimeric liver. At baseline (day 0 post-infection), mean body weights across all randomized mice were 18.0 g (19.0–21.1 g) with average serum levels of h-Alb of 10.0 mg/mL (9.2–11.9 mg/mL). Throughout the infection, all mice maintained a body weight of more than 90% of the initial level (HBV-inoculated group ranged from 90% (17.6 g) to 110% (21.6 g) to that in the control group) and an average blood h-Alb concentration of more than 10 mg/mL (HBV-inoculated group ranged from 90% (9.7 mg/mL) to 110% (11.9 mg/mL) to that in the control group).

For the ACC inhibitor efficacy study, HBV-infected mice were randomized into two different treatment groups based on body weight, blood h-Alb, and serum HBV DNA concentrations. All mice had blood h-Alb levels above 12 mg/mL and serum HBV DNA levels above 2.5x10^7^ copies/mL. Mice received an oral dose of 10 mg/kg ACC inhibitor or dosing vehicle (vehicle control) once daily for 29 days. ACC inhibitor was dosed in 0.5% sodium carboxymethylcellulose (medium viscosity), 1% w/v ethanol, 98.5% w/v 50 mM Tris buffer, pH 8 in reverse osmosis water (or equivalent). Terminal livers were collected, weighed, and separated into pieces that were flash frozen in liquid nitrogen or immersed in RNAlater solution (Life Technologies, Carlsbad, CA).

All animal work was performed by PhoenixBio Co, Ltd (Higashi-Hiroshima City, Japan). Detailed observations of general condition and individual body weights were conducted once daily throughout the in-life phase. All animal protocols were performed in accordance with the Guide for the Care and Use of Laboratory Animals and approved by the Animal Welfare Committee of Phoenix Bio Co., Ltd. All mice were housed individually and maintained in accordance with the Animal Ethics Committee of PhoenixBio (resolution #2518 and 2552). All surgery was performed under isoflurane anesthesia, and all efforts were made to minimize suffering. On Day 17, Animal ID No. 107 (vehicle-treated) was found dead. Since enlarged thymus, spleen and liver were observed at the necropsy, the cause of death in this animal is considered to be metastatic thymoma which has been reported to be a spontaneous change in the SCID mice.

### Serological and intrahepatic measurements

Whole blood h-Alb was determined using (BioMajestyTM Series JCA-BM6050, JEOL Ltd., Tokyo, Japan). Serum human alanine aminotransferase (h-ALT1) concentration was determined based on Enzyme-Linked ImmunoSorbent Assay (ELISA) developed by Institute of Immunology Co., Ltd. (Tokyo, Japan). The real-time detection PCR to measure the serum HBV DNA concentration was performed using the KUBIX HBV qPCR Kit (KUBIX Inc.) and CFX96 Touch™ Real-Time PCR Detection System. The primers and probes consisted of the following: forward primer (5’-CACATCAGGATTCCTAGGACC-3’), reverse primer (5’-AGGTTGGTGAGTGATTGGAG-3’), TaqMan probe (5’-6-FAM-CAGAGTCTAGACTCGTGGTGGACTTC-TAMRA-3’). Serum HBsAg and HBeAg concentration were determined by SRL, Inc. (Tokyo, Japan) based on Chemiluminescent Enzyme Immuno Assay (CLEIA) developed by Fujirebio. Serum and intrahepatic triglycerides analysis were performed by Skylight Biotech Inc. (Akita, Japan). Terminal plasma and liver were collected 2-hours after ACC inhibitor dose for PK analysis which was performed by Charles River Laboratories (Wilmington, MA). RNA was isolated from RNAlater®-immersed liver samples with RNeasy kit following manufacturer’s instructions (Qiagen). qRT-PCR on the targeted genes was evaluated by Taqman (Assay IDs Hs04984975_m1 (SREBF1), Hs01682761_m1 (SCD), Hs01005622_m1 (FASN), Hs01046047_m1 (ACACA) and normalized to GAPDH endogenous control (Assay ID 4310884E).

### Metabolomics

Flash frozen liver tissue samples were collected from chimeric humanized mice and delivered to Metabolon Inc. (Durham, NC, USA) for global untargeted and complex lipid panel analysis. All mice were reconstituted at same age and uninfected were also 56 days post “no infection”. A total of 1698 metabolites were identified in liver tissue samples. Samples were extracted using methanol in the presence of internal standards and split into equal parts for analysis using ultraperformance liquid chromatography coupled with high-resolution/accurate mass spectrometry (UPLC-MS/MS) and Polar LC platforms. Several internal standards were added to each experimental and process standard sample just prior to injection into the mass spectrometers to measure instrument variation. In addition, the relative standard deviation for the metabolites that were consistently measured in the CMTRX represents the total variability within the process for the actual experimental samples and the variability in quantitation of the endogenous metabolites within these samples. QC measurements were within Metabolon’s QC specifications below 6%. Metabolites were identified by their *m/z* retention time and through comparison with library entities of purified known standards. Peaks were quantified as area-under-the-curve detector ion counts. Non-adjusted *p*-values for each comparison is derived from the natural log-transformed data using Welch’s two-sample t-tests. In metabolomics, the directionality and/or pattern of biochemical changes within a pathway often point to important shifts in biological themes or diseases even if the metabolites have high adjusted *p*-values or trending *p*-values (0.05<p<0.10). Complete metabolomics data is available in S1 Table.

### RNAseq

Isolation of total cellular RNA and RNA-Seq was conducted by Expression Analysis (Durham, NC) as described previously [21]. On-column DNase I treatment was performed during RNA isolation with the RNeasy Mini Kit (Qiagen) and cDNA libraries were constructed using a TruSeq Stranded mRNA Library Prep Kit (Illumina, San Diego, CA). Pair-end sequencing was conducted using Illumina HiSeq2000 with read length of 50 nucleotides averaging approximately 30 million reads per sample. RNAseq analysis was conducted as previously described [22] and sequencing reads aligned to the human and HBV genomes by STAR [23]. The Bioconductor packages edgeR and limma were used to normalize sequence count data and conduct differential gene expression analysis [24, 25]. False discovery rate (FDR) was calculated using the Benjamini-Hochberg method. RNASeq data generated in this study were deposited in the Gene Expression Omnibus (http://www.ncbi.nlm.nih.gov/geo) with accession number GSE201037.

### HBV virion production and infection of HepG2-NTCP cells

Production of wild-type HBV virions from HepAD38 cells was performed as previously described [26]. HepG2-NTCP cells were bulk plated on collagen coated flasks at a density of 6x10^6^ cells/ T175 flask. Following a 3-day incubation at 37°C, cells were infected with genotype D (AD38) virus at 1000 GE/ overnight in the presence of 4% PEG8000 and 2.5% DMSO. HepG2-NTCP cells were washed 3 times with OPTI-MEM and maintained in Dulbecco’s modified Eagle’s medium (DMEM) supplemented with 2% FBS and 2.5% DMSO for the following 4 days. HBV-infected HepG2-NTCP cells were trypsinized, resuspended in DMEM supplemented with 2% FBS and 1% DMSO, and seeded onto serially diluted small molecule inhibitors at a 0.5% final DMSO concentration (S1 Table) in 384-well collagen coated plates at a density of 20,000 cells/well for 3 additional days.

### HBsAg and HBeAg analysis

Cell culture supernatant (3 μL) was transferred to two 1536-well plates and HBsAg and HBeAg levels subsequently measured by HTRF (Cisbio). HBsAg was measured using Terbium conjugated (XTL-17) and D2 receptor conjugated (XTL19) anti-HBsAg antibodies, while HBeAg was measured using Terbium conjugated (GWB-F19420, Genway Biotech) and D2 receptor conjugated (GWB-24DEF, Genway Biotech) anti-HBeAg antibodies. Absorbance was measured using an Envision plate reader at emissions of 665 nm and 615 nm. The ratio of 665/615 was used to plot compound dose response curves and EC_50_ values were calculated from the fit of the dose−response curves to a four-parameter equation.

### Cell viability analysis

Cells were analyzed for cell viability by addition of CellTiter Glo (Promega) to the assay plates and analyzed using an Envision plate reader (Perkin Elmer). CC_50_ values were calculated from the fit of the dose−response curves to a four-parameter equation. All CC_50_ values represent geometric mean values of a minimum of four determinations. These assays generally produced results within 3-fold of the reported mean.

### Statistical analysis

Figs 2 and 3 and Tables 1 and 2. Welch’s two-sample two-sided *t*-test was used to test whether two unknown means are different from two independent populations.

**Table 1:** Biochemical pathway analysis on HBV induced metabolic changes in free fatty acids metabolism.

Pathway	Biochemical Name	HBV/ No HBV (fold change)	*p*-value (non-adjusted)
Fatty Acid Synthesis	Malonyl carnitine	0.96	0.709
Fatty Acid Metabolism	acetyl CoA	0.72	0.549
Long Chain Saturated Fatty Acid	myristate (14:0)	1.65	0.156
	pentadecanoate (15:0)	1.18	0.470
	palmitate (16:0)	1.37	0.176
	margarate (17:0)	1.17	0.590
	stearate (18:0)	1.27	0.226
	arachidate (20:0)	1.12	0.592
Long Chain Monounsaturated Fatty Acid	palmitoleate (16:1n7)	1.69	0.123
	10-heptadecenoate (17:1n7)	1.39	0.297
	oleate/vaccenate (18:1)	1.28	0.268
	10-nonadecenoate (19:1n9)	1.23	0.591
Long Chain Polyunsaturated Fatty Acid (n3 and n6)	stearidonate (18:4n3)	1.62	0.174
	heneicosapentaenoate (21:5n3)	1.29	0.368
	docosapentaenoate (n3 DPA; 22:5n3)	1.28	0.362
	nisinate (24:6n3)	1.12	0.756
	hexadecadienoate (16:2n6)	1.58	0.147
	linoleate (18:2n6)	1.33	0.277
	linolenate [alpha or gamma; (18:3n3 or 6)]	1.57	0.189
	dihomo-linoleate (20:2n6)	1.17	0.645
	dihomo-linolenate (20:3n3 or n6)	1.20	0.410
	docosadienoate (22:2n6)	1.35	0.365

**Table 2:** Biochemical pathway analysis of HBV induced changes in cholesterol and lipid metabolites.

Pathway	Biochemical Name	HBV/ No HBV (fold change)	*p*-value (non-adjusted)
Mevalonate Metabolism	3-hydroxy-3-methylglutarate	1.56	0.018
Sterol	cholesterol	0.99	0.924
	cholesterol sulfate	0.91	0.268
	4-cholesten-3-one	1.07	0.591
	campesterol	0.81	0.598
	7-hydroxycholesterol (alpha or beta)	0.87	0.357
Phosphatidylserine (PS)	1-palmitoyl-2-arachidonoyl-GPS (16:0/20:4)	0.93	0.635
	1-stearoyl-2-oleoyl-GPS (18:0/18:1)	0.77	0.019
	1-stearoyl-2-linoleoyl-GPS (18:0/18:2)	0.81	0.018
	1-stearoyl-2-arachidonoyl-GPS (18:0/20:4)	0.93	0.520
Sphingolipid Synthesis	sphinganine	1.18	0.062
	sphingadienine	1.21	0.186
	phytosphingosine	1.10	0.265
Sphingosines	sphingosine	1.18	0.041
	sphingosine 1-phosphate	0.96	0.884
	hexadecasphingosine (d16:1)*	1.22	0.022
	heptadecasphingosine (d17:1)	1.14	0.274
Glycosyl PE	1-palmitoyl-2-arachidonoyl-glycosyl-GPE (16:0/20:4)**	2.00	0.120
	1-palmitoyl-2-docosahexaenoyl-glycosyl-GPE (16:0/22:6)**	1.96	0.292
	1-stearoyl-2-linoleoyl-glycosyl-GPE (18:0/18:2)**	1.88	0.087
	1-stearoyl-2-arachidonoyl-glycosyl-GPE (18:0/20:4)**	1.78	0.147
	1-stearoyl-2-docosahexaenoyl-glycosyl-GPE (18:0/22:6)**	2.25	0.091

* Indicates a compound that has not been confirmed based on a standard

** indicates a compound for which a standard is not available. Metabolites were identified by virtue of their recurrent nature (both chromatographic and mass spectral).

Fig 4B and 4D. Dose response curve was fit in GraphPad Prism 8.1.2. using 4 parameter variable slope dose-response model.

Fig 5C–5E. Statistical analysis between ACC inhibitor and vehicle-treated groups was performed using unpaired two-tailed t-test in GraphPad Prism 8.1.2. *, p-value ≤ 0.05.

## Results

### HBV infection modulates lipid metabolism in the livers of HBV-infected chimeric humanized mice

**Fig 1:**
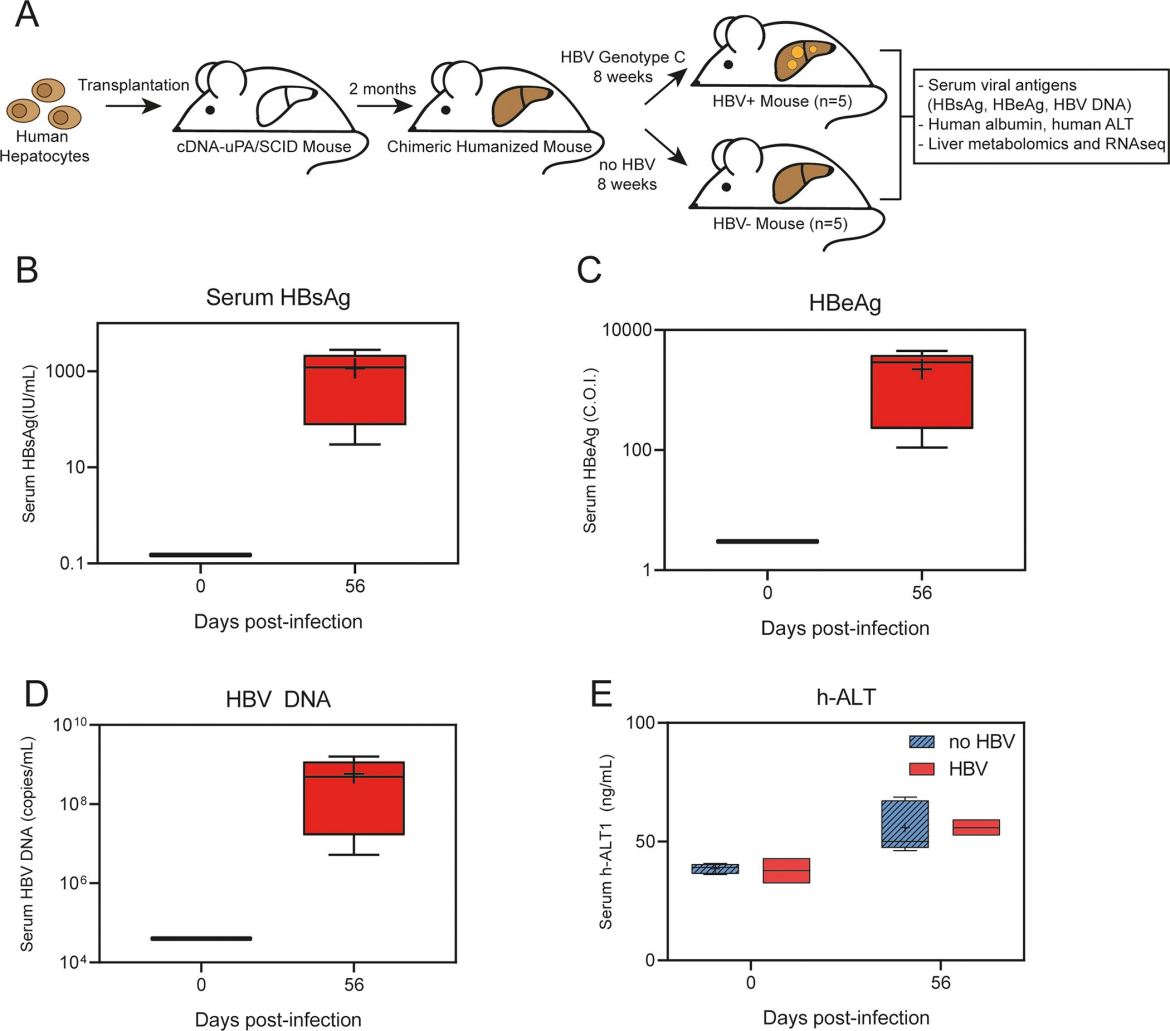
Chimeric humanized mouse model to identify host factors and pathways modulated by HBV infection. (A) Schematic overview of the study design and samples analyses. (B-D) Levels of serum viral markers at 0- and 56-days post HBV infection: HBsAg (B), HBeAg (C) and HBV DNA (D). (E) Serum levels of h-ALT in HBV-infected and non-infected mice measured at 0- and 56-days post- infection. Data is shown as box plots; the box represents the upper and lower quartile, error bars denote the max and min of distribution, the ‘+’ sign represents the mean value and the horizontal line indicates the median value.

Metabolomic and RNAseq analysis of liver tissue samples from uninfected (n = 5) and HBV-infected (n = 5) humanized chimeric liver (uPA/SCID) mice were performed to identify potential new host factors and pathways modulated by HBV infection (Fig 1A). Although immunodeficient, this model closely mimics human HBV infection as it can support a long-term persistent infection with the full HBV replication cycle [27]. Stable and complete infection of the majority of human hepatocytes was confirmed by measuring several parameters of viral infection, including serum HBsAg, HBeAg and HBV DNA. Terminal mean serum HBsAg levels were 4.5x10^2^ IU/mL (Fig 1B), serum HBeAg levels were 1.1x10^5^ cut-off index (C.O.I.) (Fig 1C), and HBV DNA was 1.5x10^8^ copies/mL (Fig 1D) at 56 days post-inoculation. As expected, no significant difference in serum h-ALT1 across HBV-inoculated and control groups as HBV is not cytopathic and there is no immune system in this model (Fig 1E).

**Fig 2:**
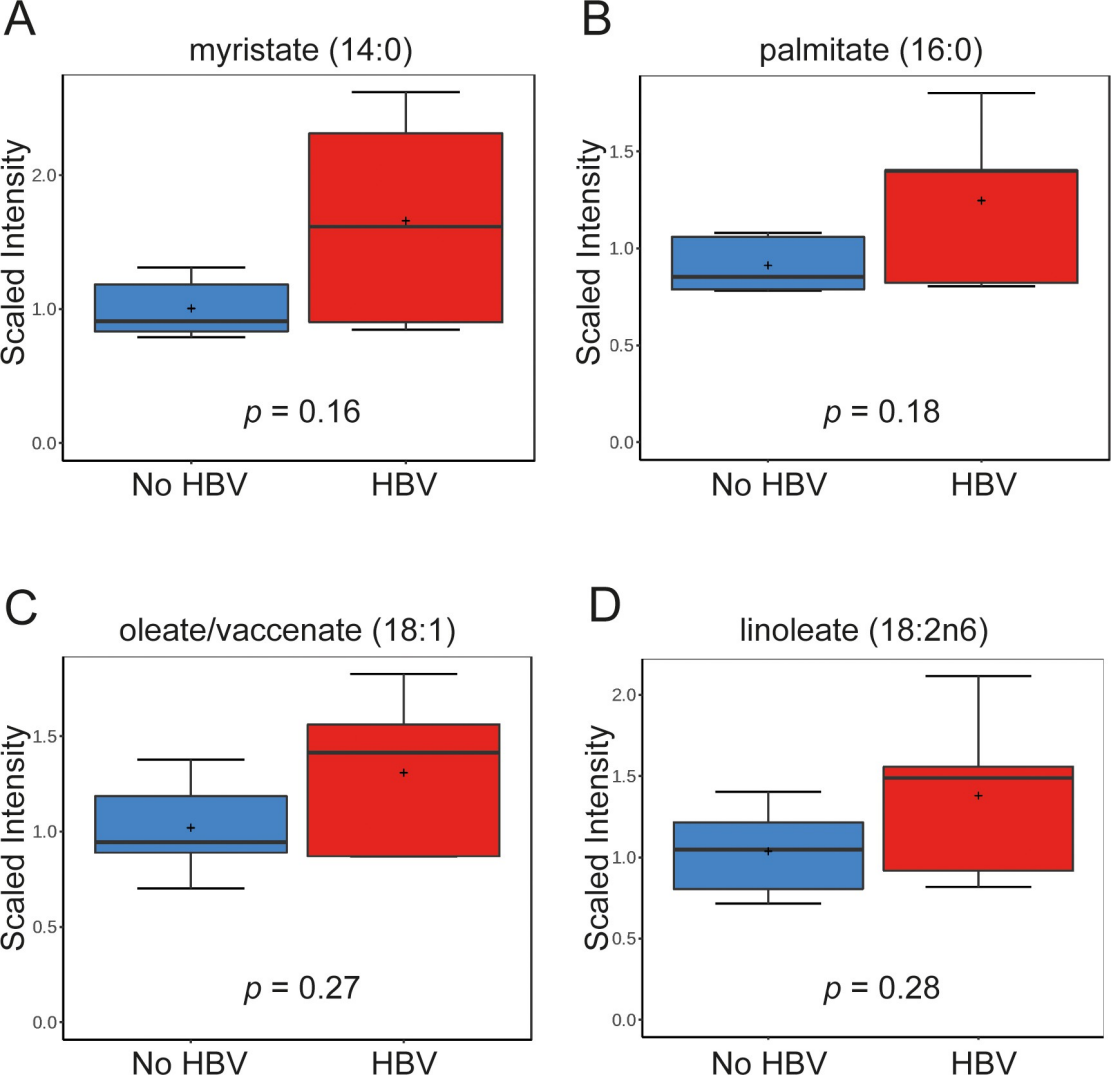
Metabolomic profiling of humanized chimeric mouse livers shows upregulation in long-chain fatty acids following HBV infection. (A-D) Intrahepatic levels of several saturated and unsaturated long chain fatty acids upregulated in HBV-infected mice compared to non-infected mice are shown: myristate (14:0) (A), palmitate (16:0) (B), oleate/vaccenate (18:1) (C) and linoleate (18:2n6) (D). Data is scaled such that the median value measured across all samples was set to 1.0. Data is shown as box plots; the box represents the upper and lower quartile, error bars denote the max and min of distribution, the ‘+’ sign represents the mean value and the horizontal line indicates the median value.

Metabolomic profiling of livers from these mice revealed HBV-dependent changes in cellular metabolites within selective lipid metabolic pathways. HBV-dependent increases were observed in several of the long-chain free fatty acids (FFA), including monounsaturated, saturated, and polyunsaturated fatty acids containing fourteen or more carbon atoms (Table 1). Even though *p*-values for individual FFA were not statistically significant, the whole group of twenty metabolites within the FFA group show an elevated trend with HBV infection which suggests dysregulation of the pathway. Specifically, HBV infection resulted in a 1.7-fold increase of myristate (C14:0), 1.4-fold increase of palmitate (C16:0), 1.3-fold increase of oleate (C18:1), and 1.3-fold increase of linoleate (C18:2) compared to non-infected samples (Fig 2A–2D).

**Fig 3:**
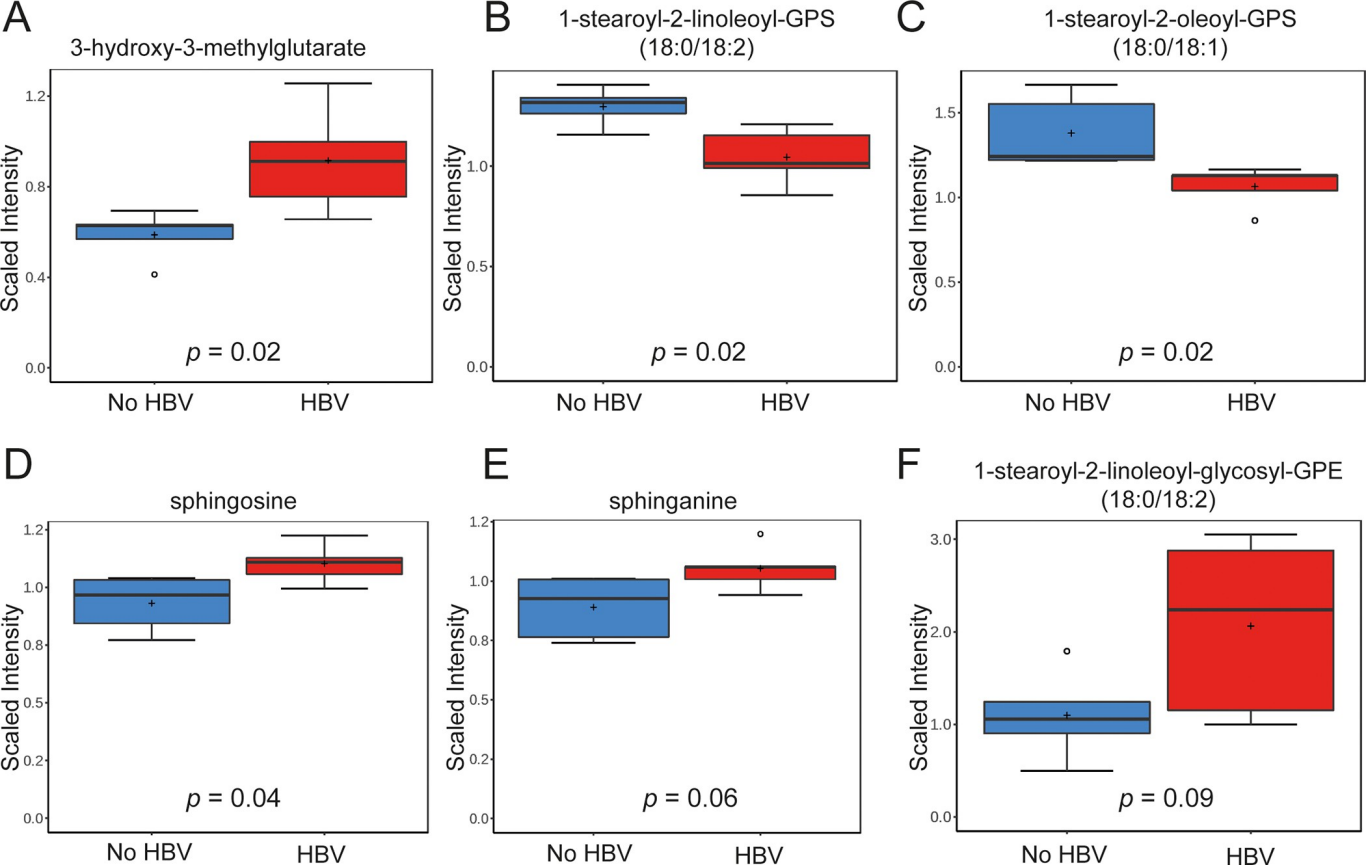
Metabolomic profiling of humanized chimeric mouse livers shows dysregulation in the levels of metabolites within mevalonate, phospholipid and sphingolipid pathways. Hepatic levels of metabolites from HBV-infected and non-infected mice within (A) mevalonate pathway (3-hydroxy-3-methylglutarate); (B-C) phospholipid pathway: 1-stearoyl-2-linoleoyl-GPS (18:0/18:2) (B) and 1-stearoyl-2-oleoyl-GPS (18:0/18:1) (C); (D-E) sphingolipid pathway: sphingosine (D) and sphinganine (E); (F) plasmalogens glycosyl-PEs (1-stearoyl-2-linoleoyl-glycosyl-GPE (18:0/18:2)). Data is scaled such that the median value measured across all samples was set to 1.0. Data is shown as box plots; the box represents the upper and lower quartile, error bars denote the max and min of distribution, the ‘+’ sign represents the mean value and the horizontal line indicates the median value.

In addition to modulation of FFA, HBV infection also resulted in statistically significant changes in the levels of metabolites within mevalonate and several other lipid pathways, including modulations in phospholipid and sphingolipid metabolites (Table 2). In particular, HBV infection resulted in a 1.6-fold increase of 3-hydroxy-3-methylglutarate (*p*-value = 0.02), 0.8-fold (*p*-value = 0.02) decreases in both 1-stearoyl-2-linoleoyl-GPS (18:0/18:2) and 1-stearoyl-2-oleoyl-GPS (18:0/18:1), and 1.2-fold increases in both sphingosine (*p*-value = 0.02) and sphinganine (*p*-value = 0.06) levels (Fig 3A–3E). Furthermore, several plasmalogens glycosyl-PEs, such as 1-stearoyl-2-linoleoyl-glycosyl-GPE (18:0/18:2), derived from a linoleic acid and an octadecanoic acid (fold change of 1.9, *p*-value = 0.09) (Fig 3F) were increased albeit did not reach statistical significance.

We also performed RNAseq on the same uninfected and HBV-infected livers from the chimeric humanized mice. No genes were found to be significantly dysregulated in the HBV-infected livers compared to the control group. The list of the top significantly dysregulated genes (*p* ≤ 0.001) without adjusting for multiple hypothesis testing is provided in S2 Table and includes changes in genes regulating cell survival and protein homeostasis. Overall, HBV infection did not lead to substantial changes in cellular gene expression or metabolomic profiles within livers of chimeric mice. However, HBV infection likely post-transcriptionally impacted several important aspects of lipid metabolism which could be central for virus infection and/or HBsAg particle formation.

### Compounds targeting enzymes within lipid biosynthetic pathways inhibit HBsAg secretion in HBV-infected HepG2-NTCP cells

**Fig 4:**
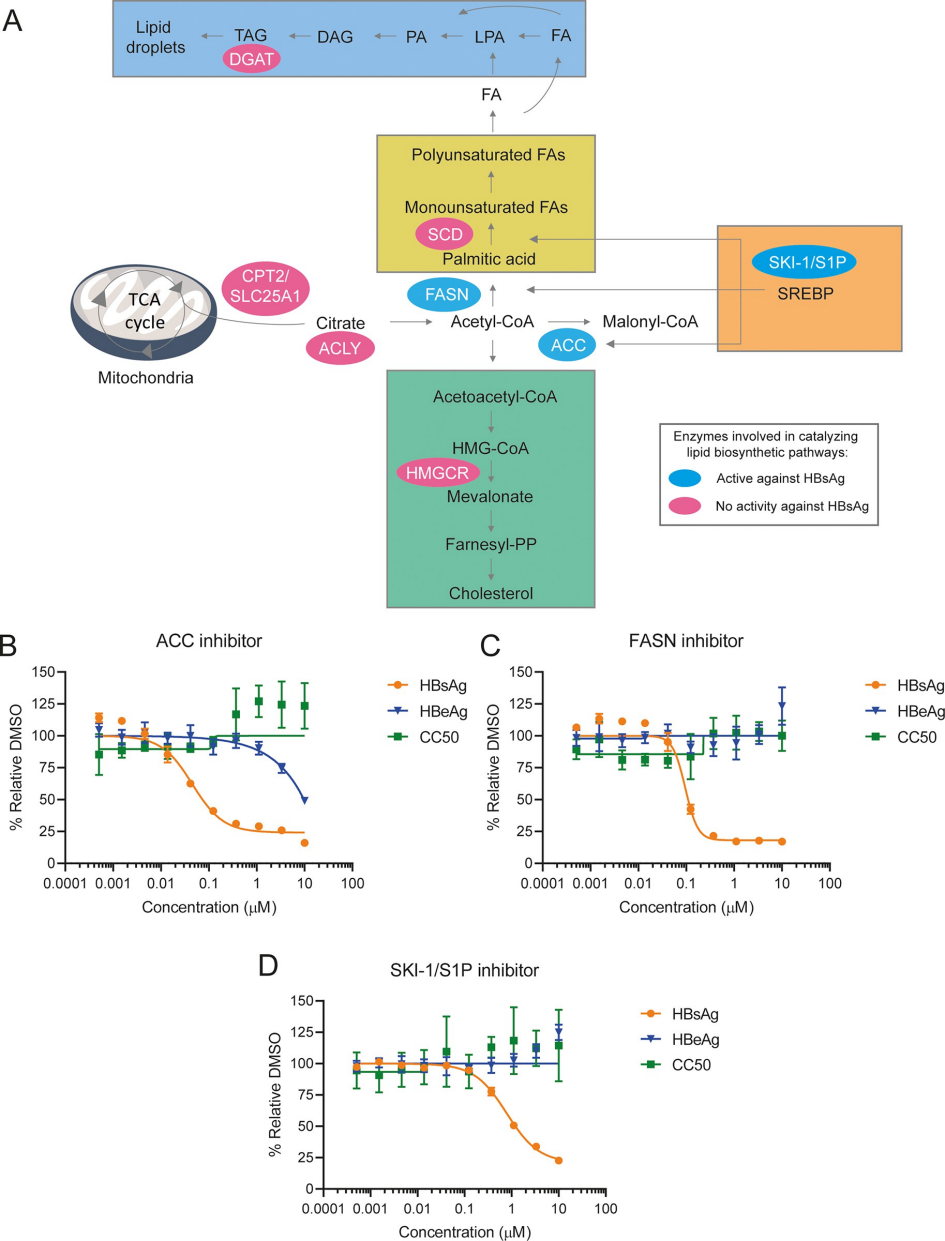
Compounds targeting enzymes within lipid biosynthetic pathways inhibit HBsAg secretion in HepG2-NTCP cells. (A) Schematic overview of the pathways involved in lipid biosynthesis, such as DNL, TG esterification and cholesterol biosynthesis. The enzymes targeted by compounds that showed anti-HBV activity in HepG2-NTCP cells are indicated with blue ellipse. The enzymes with no activity against HBV in HepG2-NTCP cells are indicated with pink ellipse. (B-D) HepG2-NTCP cells infected with HBV for three days were treated with selected compounds in an eight-point dose response. After three days of treatment, extracellular HBsAg and HBeAg levels as well as cell viability were measured. Effect of ACC (B), FASN(C) and SKI-1/S1P (D) inhibitors on extracellular HBsAg and HBeAg and cell viability in HBV-infected HepG2-NTCP cells. Results are presented as mean and standard deviation (SD).

To evaluate if perturbation of lipid biosynthetic pathways modulated by HBV infection could interfere with the HBV replication cycle, we assessed the antiviral activity of small molecules in HBV infected HepG2-NTCP cells targeting key enzymes within lipid biosynthesis. Using pre-clinical tool compounds with well-characterized mechanism of action, we targeted enzymes within DNL pathways, triglyceride esterification, and cholesterol biosynthesis (Fig 4A and S3 Table). Compounds targeting ACC, FASN and subtilisin kexin isozyme-1/site-1 protease (SKI-1/S1P) all inhibited HBsAg secretion (up to 80%) with EC_50_ values of 29, 81, and 790 nM, respectively (Fig 4B–4D). Compounds which showed HBV activity included a liver-directed allosteric inhibitor of ACC, selective inhibitor of FASN, and an active-site-directed aminopyrrolidineamide-based inhibitor of SKI-1/S1P [28, 29]. Of note, both the FASN and SKI-1/S1P inhibitors were selective for HBsAg, while inhibition of ACC also showed inhibition of HBeAg but at a higher concentration than HBsAg (Fig 4B–4D). These compounds showed only mild HBV DNA reduction (up to 40%), but no measurable activity against intracellular HBV RNA levels, intracellular HBsAg and HBV core levels (S1A and S1B Fig). No measurable cellular cytotoxicity was observed at the tested concentrations for these compounds (Fig 4B–4D). Compounds targeting other enzymes within lipid biosynthesis pathways had no anti-HBV activity (S3 Table).

### ACC inhibitor has no effect on HBV infection *in vivo*

**Fig 5:**
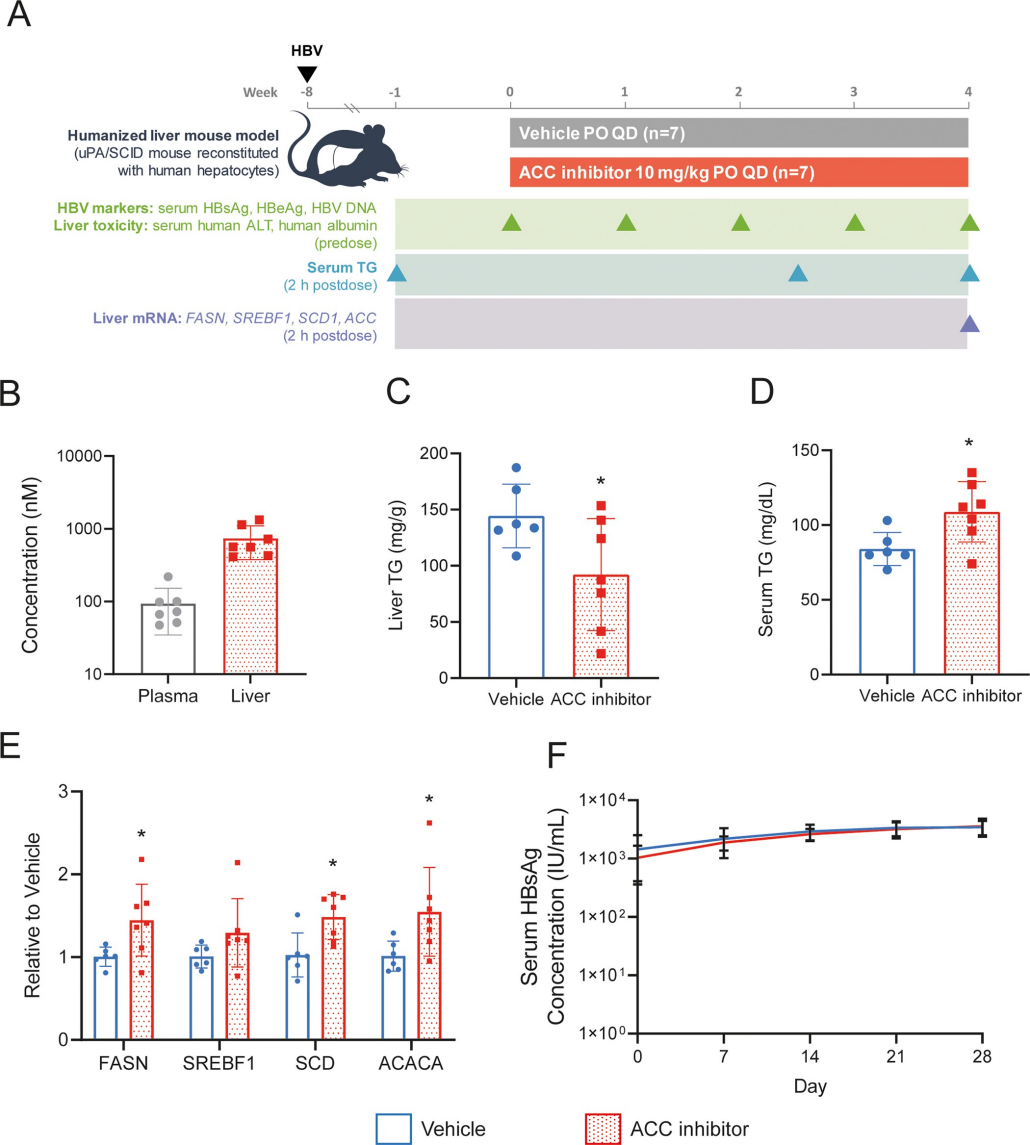
ACC inhibitor as no effect on HBV infection *in vivo*. (A) Schematic overview of ACC inhibitor efficacy study in the chimeric humanized mouse model. (B) Plasma and liver PK analysis at 2 hours post-dosing. (C) Liver triglycerides measured on day 29 of treatment. (D) Serum triglycerides measured on day 17 of treatment. (E) Liver RNA PD markers of ACC inhibitor were analyzed by qRT-PCR. (F) Longitudinal analysis of serum HBsAg levels during 4-week treatment. All data are presented as mean +/- SD. Asterisks denote *p*-value below 0.05.

Given the potency of the ACC inhibitor against HBsAg secretion in HBV-infected HepG2-NTCP cells and its previously reported on-target activity *in vivo* [30], we next performed an efficacy study in the HBV-infected chimeric humanized mouse model. Using the same batch of human hepatocytes and HBV virus used for the metabolomic and RNAseq analyses, mice (n = 7/group) were dosed orally eight weeks post HBV infection with vehicle or 10 mg/kg ACC inhibitor once daily for four weeks. HBV viral markers (HBsAg, HBeAg, and HBV DNA) and liver toxicity markers (ALT, h-Alb) were measured weekly (Fig 5A). On-target activity was evaluated by monitoring changes in triglycerides and liver mRNA expression levels of genes in the targeted pathway (Fig 5A). Firsocostat (FIR) is a clinical stage liver-targeted allosteric ACC inhibitor that has been shown to reduce hepatic fat content in patients with NASH [31]. The ACC inhibitor used here and in our *in vitro* studies is a structural analog of FIR with similar potency of DNL and palmitic acid-induced triglyceride accumulation (EC_50_ = 6.6 nM and 23 nM in HepG2 cells, respectively), and selectivity [28, 32]. Additionally, its pharmacokinetic properties in rodents replicate those of FIR in human, allowing its use as a convenient tool to mimic human FIR pharmacology in rodent models [28]. There were comparable levels of serum triglycerides (mean of 107 mg/mL per group) in both groups one week prior to compound administration (S2A Fig).

Liver exposure of the ACC inhibitor was 740 nM two hours post dosing, which is equivalent to 4.5-fold over protein-adjusted EC_50_ (paEC_50_) based on HepG2-serum free-DNL assay (Fig 5B). One mouse died in the vehicle treated group, but there was no evidence of toxicity in the mice treated with the ACC inhibitor based on body weight change, serum ALT, and h-Alb levels (S2B–S2D Fig). Statistically significant reductions in liver triglycerides (40%) (Fig 5C) and an increase in serum triglycerides (30%) (Fig 5D) confirmed on-target activity. Additionally, statistically significant increase in liver mRNA expression levels of *FASN*, *SCD* and Acetyl-CoA Carboxylase Alpha (*ACACA)* further support on-target engagement [30] (Fig 5E). Liver mRNA expression levels of *SREBF1* also increased with ACC treatment albeit did not reach statistical significance (Fig 5E). Despite the confirmed on-target activity, there was no reduction in serum HBsAg, HBeAg or HBV DNA levels throughout the study (Fig 5F and S2E and S2F Fig).

## Discussion

In search of new potential therapeutic targets, we investigated HBV-dependent metabolic and transcriptional changes utilizing the humanized chimeric liver mouse model to mimic chronic human HBV infection as closely as possible. This model is better suited for host metabolic pathways analysis compared to other HBV animal models as it can support a long-term persistent infection undergoing the full HBV replication cycle in human hepatocytes following direct inoculation. High levels of HBV DNA and antigens can be detected in the serum, cccDNA can be isolated from hepatocytes, and mice do not resolve the infection [27, 33]. However, the immune response pathways against HBV cannot be investigated as these mice are immunodeficient. Overall, the results of our analysis demonstrated that HBV establishes infection *in vivo* with only subtle changes to the host metabolome and RNA transcription profiles, consistent with RNAseq data in HBV-infected primary hepatocytes [22].

Changes induced by HBV infection in this model were largely limited to post-transcriptional modulation of lipid metabolic pathways which are potentially important for assembly and secretion of HBsAg particles. One such upregulated lipid pathway belongs to a group of long-chain FFAs, including monounsaturated, saturated, and polyunsaturated fatty acids. These findings are consistent with the previous literature showing increases in long-chain FFAs in primary rat hepatocytes infected with recombinant adenovirus expressing the HBV genome, and increased lipid biosynthesis through sterol regulatory element-binding protein 1c (SREBP1c) in HBV transgenic mice [34–36]. Furthermore, HBV-dependent increases in total fatty acid content was observed in a stable HBV-producing cell line (HepG2.2.15) compared to the parental control [37]. As the rapid HBsAg particle assembly and secretion is accompanied by the continuous replenishment of cellular membranes [38, 39], the observed increases in fatty acid synthesis and metabolism likely support this.

In addition to increased FFAs, we also identified HBV-dependent alterations of the mevalonate pathway with specific downregulation of 3-hydroxy-3-methylglutarate. Thought to be an antilipemic agent, 3-hydroxy-3-methylglutarate lowers cholesterol, triglycerides, serum beta-lipoproteins, and phospholipids by interfering with the enzymatic steps involved in the conversion of acetate to hydroxymethylglutaryl coenzyme A as well as inhibiting the activity of the cholesterol rate-limiting enzyme, hydroxymethylglutaryl CoA reductase (HMGCR) [40, 41]. Together, these results suggest HBV-dependent increases in cholesterol demand. Indeed, depletion of cellular cholesterol *in vitro* using lovastatin significantly reduced HBsAg secretion in Hep3B cells [16]. In our study we were not able to reduce HBsAg levels in *de novo* infected HepG2-NTCP cells with HMGCR inhibitor, though this discrepancy is likely due to the differences in the cell culture media as it has previously been shown that effects of HMGCR on HBsAg required serum starvation. Another recent report supporting the importance of cholesterol for HBV infection showed that inhibition of acyl-CoA:cholesterol acyltransferase (ACAT) has antiviral activity against both secreted HBV DNA and HBsAg in HepG2-NTCP cells, as well as boosting protective anti-HBV and anti-hepatocellular carcinoma T cells [42].

Our metabolomic analysis indicated that HBV infection results in modulation of several phospholipid and sphingolipid metabolites in the liver. Together, these shifts in phospholipid and sphingolipid levels may reflect changes in cellular membrane composition and/or integrity, or lipoprotein particle composition and abundance. The latter hypothesis is supported by the glycosyl-PE shifts which is a crucial player in lipoprotein secretion [43, 44]. Since phospholipids constitute the surface layer of lipid droplets (LD), their reduction in the liver may decrease the storage capacity of LDs. This raises the possibility that HBV infection might alter the breakdown of hepatic phospholipids in LDs resulting in an altered lipid storage capacity of the liver. Indeed, this could be one reason why cholesterol and sphingolipid synthesis are elevated in the HBV-infected mice. In support of this hypothesis, a study demonstrated that the content of intracellular TGs and the average size of a single LD were significantly reduced in HBV-infected or transfected cells compared to control cells. This reduction was due to decreased levels of proteins involved in LD expansion and lipid storage [45]. Significant changes in phospholipids and sphingolipids have also been observed in CHB patients [46–48]. Additionally, since sphingosine and sphinganine are bioactive lipids that are involved in lipid signaling, a rise in these two metabolites after HBV infection may warrant further investigation.

Given the observed HBV-dependent increases of FFA, we sought to investigate whether modulation of lipid biosynthesis pathways can inhibit viral infection and/or HBsAg particle formation. As primary human hepatocytes require lipid supplements in their media for survival *in vitro*, we utilized HBV-infected HepG2-NTCP cells for this profiling. We observed inhibition of enzymes involved in DNL, such as ACC, FASN, and SKI-1/S1P, selectively reduced extracellular HBsAg levels. SKI-1/S1P is one of two proteases required to cleave and activate SREBP precursors, while FASN and ACC are direct targets of SREBP1c directly involved in the biosynthesis of fatty acids [29, 49]. Inhibition of these enzymes has been previously reported to be effective *in vitro* against human RNA viruses, including Hepatitis C, Dengue, West Nile, and Zika, underlining the importance of lipid pathways for viral infection [50–53].

ACC inhibitors are being evaluated in several clinical studies in patients with non-alcoholic steatohepatitis (NASH). Phase 1b clinical studies in F1-F3 NASH patients with FIR, an allosteric liver-targeted inhibitor of both ACC isoforms (ACC1 and ACC2), demonstrated a reduction in hepatic fat content and markers of liver injury [31, 54]. ACC1 and ACC2 catalyze the conversion of acetyl coenzyme A to malonyl-CoA [55, 56]. Cytosolic ACC1 generates a pool of malonyl-CoA used by FASN to generate palmitate as a part of DNL, which is further incorporated into multiple lipid species including ceramides, diacylglycerides, and triglycerides. ACC2, located at the mitochondrial membrane, supplies a local pool of malonyl-CoA to allosterically inhibit carnitine palmitoyl transferase 1, the rate-limiting enzyme that imports medium- and long-chain fatty acids into the mitochondria for β-oxidation [56]. Together, the two isoforms of ACC increase the total lipid burden by coupling increased lipid production via DNL with decreased lipid oxidation via mitochondrial β-oxidation. While potent inhibition of HBsAg secretion was observed in the cell culture system with an inhibitor of ACC1/2, no activity was observed in a four-week efficacy study in HBV-infected liver chimeric mice, despite confirmed on-target activity. The dose selected for our in vivo studies was intended to mimic the partial target inhibition (~70% DNL inhibition) affording ~30% liver fat reduction observed clinically [31, 54]. Consistent with this, Fig 5C shows partial inhibition of liver triglycerides with the 10 mg/kg dose in the HBV-infected chimeric humanized mice. It is possible that dependence of HBsAg on the DNL pathway may be less relevant *in vivo* where lipid contributions towards HBsAg production may be derived from other sources such as lipolysis or diet. Thus, a combination treatment targeting several lipid metabolic pathways may be necessary to reduce HBsAg levels *in vivo*.

Overall, our study identified several novel lipid metabolic pathways dysregulated during HBV infection in the HBV-infected liver chimeric mouse model. Modulation of these lipid pathways offer new potential avenues for HBV cure. We show that the levels of FFAs are increased in the livers following HBV infection and targeting lipogenic enzymes inhibits HBsAg secretion in a cell culture system. The discrepancy between the anti-HBsAg effect of the ACC inhibitor *in vitro* and *in vivo* models warrants further investigation.

## Supporting information

S1 Fig ACC, FASN and SKI-1/S1P inhibitors activity against HBV infection. (A, B) HepG2-NTCP cells infected with HBV for three days were treated with selected compounds in an eight-point dose response. After three days of treatment, extracellular HBV DNA, intracellular HBV RNA and intracellular HBsAg levels were measured. EC_50_ values are shown in the table. (B) Fixed cells were stained for cell nuclei using DAPI stain (blue), HBsAg (green) and HBV core protein (yellow). Representative images of HepG2-NTCP cells treated with selected compounds at 10 μM obtained with confocal Opera Phenix with a 20x objective are shown. Scale bar represents 100 μm.
(TIF)

S2 Fig ACC inhibitor efficacy study in the chimeric humanized mouse model. (A-F) Laboratory parameters measured during the study in ACC inhibitor- and vehicle-treated groups. Serum triglycerides levels 7 days before treatment (A); body weights (B); serum ALT activity (C); h-Alb concentration (D); serum HBeAg concentration (E); serum HBV DNA (F). Data is shown as mean +/- SD.
(TIF)

S1 Table HBV metabolomics data tables. (XLSX)

S2 Table Top dysregulated genes in the HBV-infected livers from chimeric humanized mice compared to non-infected mice based on RNAseq analysis. (PDF)

S3 Table Anti-HBV activity and toxicity of compounds modulating lipid pathways. (PDF)

S1 File Supporting materials and methods. (PDF)

## List of abbreviations

HBV: Hepatitis B virus
HBsAg: HBV surface antigen
ACC: acetyl-CoA carboxylase
CHB: chronic Hepatitis B
NA: Nucleos(t)ide analogue
FA: fatty acids
DNL: de novo lipogenesis
FASN: FA synthase
DGAT: diacylglycerol acyltransferase
SCD: stearoyl-CoA desaturase
SREBPs: sterol regulatory element binding proteins
C.O.I: cut-off index
h-ALT1: human alanine aminotransferase
FFA: free fatty acids
SKI-1/S1P: subtilisin kexin isozyme-1/site-1 protease
h-Alb: human albumin
FIR: Firsocostat

## References

[1] WHO. Global hepatitis report, 2017. Geneva 2017: World Health Organization; 2017.

[2] Burton AR, Pallett LJ, McCoy LE, Suveizdyte K, Amin OE, Swadling L, et al. Circulating and intrahepatic antiviral B cells are defective in hepatitis B. J Clin Invest. 2018;128(10):4588–603. Epub 2018/08/10. doi: 10.1172/JCI121960 ; PubMed Central PMCID: PMC6159997. 30091725 PMC6159997

[3] Salimzadeh L, Le Bert N, Dutertre CA, Gill US, Newell EW, Frey C, et al. PD-1 blockade partially recovers dysfunctional virus-specific B cells in chronic hepatitis B infection. J Clin Invest. 2018;128(10):4573–87. Epub 2018/08/08. doi: 10.1172/JCI121957 ; PubMed Central PMCID: PMC6159957. 30084841 PMC6159957

[4] Le Bert N, Salimzadeh L, Gill US, Dutertre CA, Facchetti F, Tan A, et al. Comparative characterization of B cells specific for HBV nucleocapsid and envelope proteins in patients with chronic hepatitis B. J Hepatol. 2020;72(1):34–44. Epub 2019/07/28. doi: 10.1016/j.jhep.2019.07.015 . 31348999

[5] Michler T, Kosinska AD, Festag J, Bunse T, Su J, Ringelhan M, et al. Knockdown of Virus Antigen Expression Increases Therapeutic Vaccine Efficacy in High-Titer Hepatitis B Virus Carrier Mice. Gastroenterology. 2020;158(6):1762–75 e9. Epub 2020/02/01. doi: 10.1053/j.gastro.2020.01.032 . 32001321

[6] Nassal M. HBV cccDNA: viral persistence reservoir and key obstacle for a cure of chronic hepatitis B. Gut. 2015;64(12):1972–84. doi: 10.1136/gutjnl-2015-309809 Epub 2015 Jun 5. 26048673

[7] Tu T, Budzinska MA, Shackel NA, Urban S. HBV DNA Integration: Molecular Mechanisms and Clinical Implications. Viruses. 2017;9(4). Epub 2017/04/11. doi: 10.3390/v9040075 ; PubMed Central PMCID: PMC5408681. 28394272 PMC5408681

[8] Gavilanes F, Gonzalez-Ros JM, Peterson DL. Structure of hepatitis B surface antigen. Characterization of the lipid components and their association with the viral proteins. J Biol Chem. 1982;257(13):7770–7. Epub 1982/07/10. . 7085648

[9] Simon K, Lingappa VR, Ganem D. Secreted hepatitis B surface antigen polypeptides are derived from a transmembrane precursor. J Cell Biol. 1988;107(6 Pt 1):2163–8. doi: 10.1083/jcb.107.6.2163 3198683 PMC2115684

[10] Seto WK, Lo YR, Pawlotsky JM, Yuen MF. Chronic hepatitis B virus infection. Lancet. 2018;392(10161):2313–24. Epub 2018/11/30. doi: 10.1016/S0140-6736(18)31865-8 . 30496122

[11] Kim GA, Lim YS, An J, Lee D, Shim JH, Kim KM, et al. HBsAg seroclearance after nucleoside analogue therapy in patients with chronic hepatitis B: clinical outcomes and durability. Gut. 2014;63(8):1325–32. Epub 2013/10/29. doi: 10.1136/gutjnl-2013-305517 . 24162593

[12] Zoulim F, Durantel D. Antiviral therapies and prospects for a cure of chronic hepatitis B. Cold Spring Harb Perspect Med. 2015;5(4). doi: 10.1101/cshperspect.a021501 25833942 PMC4382723

[13] Hyrina A, Jones C, Chen D, Clarkson S, Cochran N, Feucht P, et al. A Genome-wide CRISPR Screen Identifies ZCCHC14 as a Host Factor Required for Hepatitis B Surface Antigen Production. Cell Reports. 2019;29(10):2970–8.e6. doi: 10.1016/j.celrep.2019.10.113 31801065

[14] Zhang J, Ling N, Lei Y, Peng M, Hu P, Chen M. Multifaceted Interaction Between Hepatitis B Virus Infection and Lipid Metabolism in Hepatocytes: A Potential Target of Antiviral Therapy for Chronic Hepatitis B. Front Microbiol. 2021;12:636897. Epub 2021/03/30. doi: 10.3389/fmicb.2021.636897 ; PubMed Central PMCID: PMC7991784. 33776969 PMC7991784

[15] Liou JW, Mani H, Yen JH. Viral Hepatitis, Cholesterol Metabolism, and Cholesterol-Lowering Natural Compounds. Int J Mol Sci. 2022;23(7). Epub 2022/04/13. doi: 10.3390/ijms23073897 . 35409259 PMC8999150

[16] Lin YL, Shiao MS, Mettling C, Chou CK. Cholesterol requirement of hepatitis B surface antigen (HBsAg) secretion. Virology. 2003;314(1):253–60. Epub 2003/10/01. doi: 10.1016/s0042-6822(03)00403-3 . 14517078

[17] Fabbrini E, Sullivan S, Klein S. Obesity and nonalcoholic fatty liver disease: biochemical, metabolic, and clinical implications. Hepatology. 2010;51(2):679–89. Epub 2009/12/31. doi: 10.1002/hep.23280 ; PubMed Central PMCID: PMC3575093. 20041406 PMC3575093

[18] Musso G, Gambino R, Cassader M. Recent insights into hepatic lipid metabolism in non-alcoholic fatty liver disease (NAFLD). Prog Lipid Res. 2009;48(1):1–26. Epub 2008/10/01. doi: 10.1016/j.plipres.2008.08.001 . 18824034

[19] Tsuge M, Hiraga N, Takaishi H, Noguchi C, Oga H, Imamura M, et al. Infection of human hepatocyte chimeric mouse with genetically engineered hepatitis B virus. Hepatology. 2005;42(5):1046–54. Epub 2005/10/27. doi: 10.1002/hep.20892 . 16250045

[20] Tateno C, Kawase Y, Tobita Y, Hamamura S, Ohshita H, Yokomichi H, et al. Generation of Novel Chimeric Mice with Humanized Livers by Using Hemizygous cDNA-uPA/SCID Mice. PLoS One. 2015;10(11):e0142145. Epub 2015/11/05. doi: 10.1371/journal.pone.0142145 ; PubMed Central PMCID: PMC4633119. 26536627 PMC4633119

[21] Menne S, Tumas DB, Liu KH, Thampi L, AlDeghaither D, Baldwin BH, et al. Sustained efficacy and seroconversion with the Toll-like receptor 7 agonist GS-9620 in the Woodchuck model of chronic hepatitis B. J Hepatol. 2015;62(6):1237–45. Epub 2015/01/07. doi: 10.1016/j.jhep.2014.12.026 ; PubMed Central PMCID: PMC4439359. 25559326 PMC4439359

[22] Niu C, Livingston CM, Li L, Beran RK, Daffis S, Ramakrishnan D, et al. The Smc5/6 Complex Restricts HBV when Localized to ND10 without Inducing an Innate Immune Response and Is Counteracted by the HBV X Protein Shortly after Infection. PLoS One. 2017;12(1):e0169648. Epub 2017/01/18. doi: 10.1371/journal.pone.0169648 ; PubMed Central PMCID: PMC5240991 does not alter our adherence to PLOS ONE policies on sharing data and materials. 28095508 PMC5240991

[23] Dobin A, Davis CA, Schlesinger F, Drenkow J, Zaleski C, Jha S, et al. STAR: ultrafast universal RNA-seq aligner. Bioinformatics. 2013;29(1):15–21. Epub 2012/10/30. doi: 10.1093/bioinformatics/bts635 ; PubMed Central PMCID: PMC3530905. 23104886 PMC3530905

[24] Robinson MD, McCarthy DJ, Smyth GK. edgeR: a Bioconductor package for differential expression analysis of digital gene expression data. Bioinformatics. 2010;26(1):139–40. Epub 2009/11/17. doi: 10.1093/bioinformatics/btp616 ; PubMed Central PMCID: PMC2796818. 19910308 PMC2796818

[25] Ritchie ME, Phipson B, Wu D, Hu Y, Law CW, Shi W, et al. limma powers differential expression analyses for RNA-sequencing and microarray studies. Nucleic Acids Res. 2015;43(7):e47. Epub 2015/01/22. doi: 10.1093/nar/gkv007 ; PubMed Central PMCID: PMC4402510. 25605792 PMC4402510

[26] Decorsiere A, Mueller H, van Breugel PC, Abdul F, Gerossier L, Beran RK, et al. Hepatitis B virus X protein identifies the Smc5/6 complex as a host restriction factor. Nature. 2016;531(7594):386–9. Epub 2016/03/18. doi: 10.1038/nature17170 . 26983541

[27] Chayama K, Hayes CN, Hiraga N, Abe H, Tsuge M, Imamura M. Animal model for study of human hepatitis viruses. J Gastroenterol Hepatol. 2011;26(1):13–8. Epub 2010/12/24. doi: 10.1111/j.1440-1746.2010.06470.x . 21175788

[28] Bates J, Vijayakumar A, Ghoshal S, Marchand B, Yi S, Kornyeyev D, et al. Acetyl-CoA carboxylase inhibition disrupts metabolic reprogramming during hepatic stellate cell activation. J Hepatol. 2020;73(4):896–905. Epub 2020/05/08. doi: 10.1016/j.jhep.2020.04.037 . 32376414

[29] Hawkins JL, Robbins MD, Warren LC, Xia D, Petras SF, Valentine JJ, et al. Pharmacologic inhibition of site 1 protease activity inhibits sterol regulatory element-binding protein processing and reduces lipogenic enzyme gene expression and lipid synthesis in cultured cells and experimental animals. J Pharmacol Exp Ther. 2008;326(3):801–8. Epub 2008/06/26. doi: 10.1124/jpet.108.139626 . 18577702

[30] Goedeke L, Bates J, Vatner DF, Perry RJ, Wang T, Ramirez R, et al. Acetyl-CoA Carboxylase Inhibition Reverses NAFLD and Hepatic Insulin Resistance but Promotes Hypertriglyceridemia in Rodents. Hepatology. 2018;68(6):2197–211. Epub 2018/05/24. doi: 10.1002/hep.30097 ; PubMed Central PMCID: PMC6251774. 29790582 PMC6251774

[31] Lawitz EJ, Coste A, Poordad F, Alkhouri N, Loo N, McColgan BJ, et al. Acetyl-CoA Carboxylase Inhibitor GS-0976 for 12 Weeks Reduces Hepatic De Novo Lipogenesis and Steatosis in Patients With Nonalcoholic Steatohepatitis. Clin Gastroenterol Hepatol. 2018;16(12):1983–91 e3. Epub 2018/05/01. doi: 10.1016/j.cgh.2018.04.042 . 29705265

[32] Okesli-Armlovich A, Kusam S, Marchand B, Vijayakumar A, Trevaskis J, Bates J. Evaluating ACC inhibitor combinations using fatty acid oxidation and lipid content assays in human hepatocyte cell lines. Hepatology. 2020;72(S1):131A–1159A. doi: 10.1002/hep.31579

[33] Ishida Y, Chung TL, Imamura M, Hiraga N, Sen S, Yokomichi H, et al. Acute hepatitis B virus infection in humanized chimeric mice has multiphasic viral kinetics. Hepatology. 2018;68(2):473–84. Epub 2018/03/25. doi: 10.1002/hep.29891 ; PubMed Central PMCID: PMC6097938. 29572897 PMC6097938

[34] Kim KH, Shin HJ, Kim K, Choi HM, Rhee SH, Moon HB, et al. Hepatitis B virus X protein induces hepatic steatosis via transcriptional activation of SREBP1 and PPARgamma. Gastroenterology. 2007;132(5):1955–67. Epub 2007/05/09. doi: 10.1053/j.gastro.2007.03.039 . 17484888

[35] Hajjou M, Norel R, Carver R, Marion P, Cullen J, Rogler LE, et al. cDNA microarray analysis of HBV transgenic mouse liver identifies genes in lipid biosynthetic and growth control pathways affected by HBV. J Med Virol. 2005;77(1):57–65. Epub 2005/07/21. doi: 10.1002/jmv.20427 . 16032730

[36] Lamontagne RJ, Casciano JC, Bouchard MJ. A broad investigation of the HBV-mediated changes to primary hepatocyte physiology reveals HBV significantly alters metabolic pathways. Metabolism. 2018;83:50–9. Epub 2018/02/08. doi: 10.1016/j.metabol.2018.01.007 ; PubMed Central PMCID: PMC5960616. 29410347 PMC5960616

[37] Li H, Zhu W, Zhang L, Lei H, Wu X, Guo L, et al. The metabolic responses to hepatitis B virus infection shed new light on pathogenesis and targets for treatment. Sci Rep. 2015;5:8421. Epub 2015/02/13. doi: 10.1038/srep08421 ; PubMed Central PMCID: PMC4325332. 25672227 PMC4325332

[38] Satoh O, Umeda M, Imai H, Tunoo H, Inoue K. Lipid composition of hepatitis B virus surface antigen particles and the particle-producing human hepatoma cell lines. J Lipid Res. 1990;31(7):1293–300. 2169517

[39] Yang F, Yan S, He Y, Wang F, Song S, Guo Y, et al. Expression of hepatitis B virus proteins in transgenic mice alters lipid metabolism and induces oxidative stress in the liver. J Hepatol. 2008;48(1):12–9. Epub 2007/11/27. doi: 10.1016/j.jhep.2007.06.021 . 18037187

[40] Kotyla P. The role of 3-hydroxy-3-methylglutaryl coenzyme a reductase inhibitors (statins) in modern rheumatology. Ther Adv Musculoskelet Dis. 2010;2(5):257–69. doi: 10.1177/1759720X10384307 ; PubMed Central PMCID: PMC3383511. 22870452 PMC3383511

[41] Igel M, Sudhop T, von Bergmann K. Pharmacology of 3-hydroxy-3-methylglutaryl-coenzyme A reductase inhibitors (statins), including rosuvastatin and pitavastatin. J Clin Pharmacol. 2002;42(8):835–45. doi: 10.1177/009127002401102731 . 12162466

[42] Schmidt NM, Wing PAC, Diniz MO, Pallett LJ, Swadling L, Harris JM, et al. Targeting human Acyl-CoA:cholesterol acyltransferase as a dual viral and T cell metabolic checkpoint. Nat Commun. 2021;12(1):2814. Epub 2021/05/16. doi: 10.1038/s41467-021-22967-7 ; PubMed Central PMCID: PMC8121939. 33990561 PMC8121939

[43] Vance JE. Phosphatidylserine and phosphatidylethanolamine in mammalian cells: two metabolically related aminophospholipids. J Lipid Res. 2008;49(7):1377–87. Epub 2008/01/22. doi: 10.1194/jlr.R700020-JLR200 . 18204094

[44] Horl G, Wagner A, Cole LK, Malli R, Reicher H, Kotzbeck P, et al. Sequential synthesis and methylation of phosphatidylethanolamine promote lipid droplet biosynthesis and stability in tissue culture and in vivo. J Biol Chem. 2011;286(19):17338–50. Epub 2011/04/02. doi: 10.1074/jbc.M111.234534 ; PubMed Central PMCID: PMC3089575. 21454708 PMC3089575

[45] Yasumoto J, Kasai H, Yoshimura K, Otoguro T, Watashi K, Wakita T, et al. Hepatitis B virus prevents excessive viral production via reduction of cell death-inducing DFF45-like effectors. J Gen Virol. 2017;98(7):1762–73. Epub 2017/07/27. doi: 10.1099/jgv.0.000813 . 28745269

[46] Huang Q, Lei H, Ding L, Wang Y. Stimulated phospholipid synthesis is key for hepatitis B virus replications. Sci Rep. 2019;9(1):12989. Epub 2019/09/12. doi: 10.1038/s41598-019-49367-8 ; PubMed Central PMCID: PMC6736851. 31506451 PMC6736851

[47] Schoeman JC, Hou J, Harms AC, Vreeken RJ, Berger R, Hankemeier T, et al. Metabolic characterization of the natural progression of chronic hepatitis B. Genome Med. 2016;8(1):64. Epub 2016/06/12. doi: 10.1186/s13073-016-0318-8 ; PubMed Central PMCID: PMC4902991. 27286979 PMC4902991

[48] Qu F, Zheng SJ, Liu S, Wu CS, Duan ZP, Zhang JL. Serum sphingolipids reflect the severity of chronic HBV infection and predict the mortality of HBV-acute-on-chronic liver failure. PLoS One. 2014;9(8):e104988. Epub 2014/08/20. doi: 10.1371/journal.pone.0104988 ; PubMed Central PMCID: PMC4138167. 25136927 PMC4138167

[49] Alves-Bezerra M, Cohen DE. Triglyceride Metabolism in the Liver. Compr Physiol. 2017;8(1):1–8. Epub 2018/01/23. doi: 10.1002/cphy.c170012 ; PubMed Central PMCID: PMC6376873. 29357123 PMC6376873

[50] Hyrina A, Meng F, McArthur SJ, Eivemark S, Nabi IR, Jean F. Human Subtilisin Kexin Isozyme-1 (SKI-1)/Site-1 Protease (S1P) regulates cytoplasmic lipid droplet abundance: A potential target for indirect-acting anti-dengue virus agents. PLoS One. 2017;12(3):e0174483. Epub 2017/03/25. doi: 10.1371/journal.pone.0174483 ; PubMed Central PMCID: PMC5365115. 28339489 PMC5365115

[51] Olmstead AD, Knecht W, Lazarov I, Dixit SB, Jean F. Human subtilase SKI-1/S1P is a master regulator of the HCV Lifecycle and a potential host cell target for developing indirect-acting antiviral agents. PLoS Pathog. 2012;8(1):e1002468. Epub 2012/01/14. doi: 10.1371/journal.ppat.1002468 ; PubMed Central PMCID: PMC3252376. 22241994 PMC3252376

[52] Samsa MM, Mondotte JA, Iglesias NG, Assuncao-Miranda I, Barbosa-Lima G, Da Poian AT, et al. Dengue virus capsid protein usurps lipid droplets for viral particle formation. PLoS Pathog. 2009;5(10):e1000632. Epub 2009/10/24. doi: 10.1371/journal.ppat.1000632 ; PubMed Central PMCID: PMC2760139. 19851456 PMC2760139

[53] Jimenez de Oya N, Esler WP, Huard K, El-Kattan AF, Karamanlidis G, Blazquez AB, et al. Targeting host metabolism by inhibition of acetyl-Coenzyme A carboxylase reduces flavivirus infection in mouse models. Emerg Microbes Infect. 2019;8(1):624–36. Epub 2019/04/20. doi: 10.1080/22221751.2019.1604084 ; PubMed Central PMCID: PMC6493301. 30999821 PMC6493301

[54] Loomba R, Kayali Z, Noureddin M, Ruane P, Lawitz EJ, Bennett M, et al. GS-0976 Reduces Hepatic Steatosis and Fibrosis Markers in Patients With Nonalcoholic Fatty Liver Disease. Gastroenterology. 2018;155(5):1463–73 e6. Epub 2018/07/31. doi: 10.1053/j.gastro.2018.07.027 ; PubMed Central PMCID: PMC6318218. 30059671 PMC6318218

[55] Brownsey RW, Zhande R, Boone AN. Isoforms of acetyl-CoA carboxylase: structures, regulatory properties and metabolic functions. Biochem Soc Trans. 1997;25(4):1232–8. Epub 1998/02/05. doi: 10.1042/bst0251232 . 9449982

[56] McGarry JD, Mannaerts GP, Foster DW. A possible role for malonyl-CoA in the regulation of hepatic fatty acid oxidation and ketogenesis. J Clin Invest. 1977;60(1):265–70. Epub 1977/07/01. doi: 10.1172/JCI108764 ; PubMed Central PMCID: PMC372365. 874089 PMC372365

