# Supplementary figures and images for: Targeting lipid biosynthesis pathways for hepatitis B virus cure
Source: PLoS One. 2022 Aug 4;17(8):e0270273. doi: 10.1371/journal.pone.0270273 (PMC9352027; doi:10.1371/journal.pone.0270273)

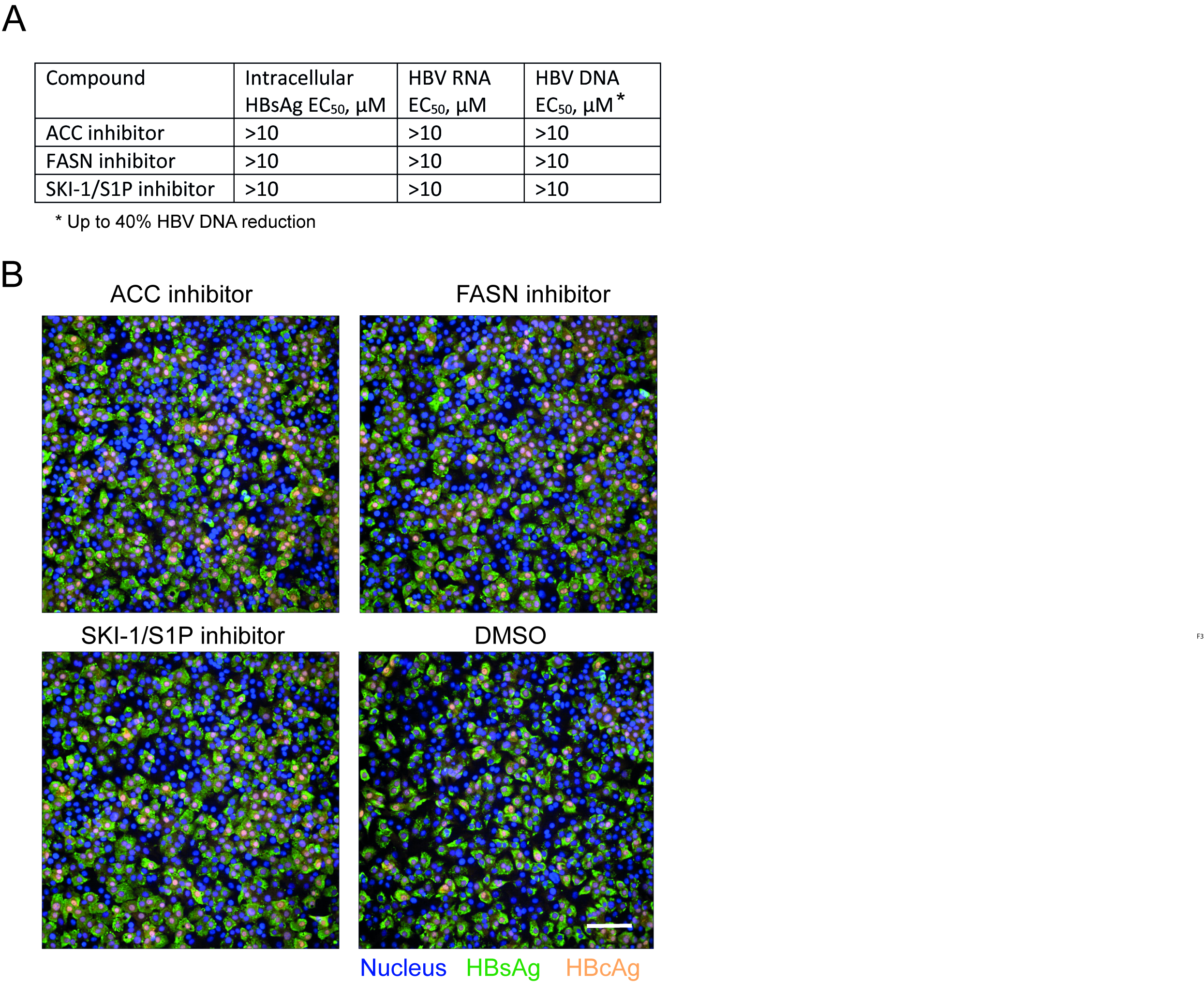

Supplement: S1 Fig — (A, B) HepG2-NTCP cells infected with HBV for three days were treated with selected compounds in an eight-point dose response. After three days of treatment, extracellular HBV DNA, intracellular HBV RNA and intracellular HBsAg levels were measured. EC50 values are shown in the table. (B) Fixed cells were stained for cell nuclei using DAPI stain (blue), HBsAg (green) and HBV core protein (yellow). Representative images of HepG2-NTCP cells treated with selected compounds at 10 μM obtained with confocal Opera Phenix with a 20x objective are shown. Scale bar represents 100 μm. (TIF) [file pone.0270273.s001.tif]

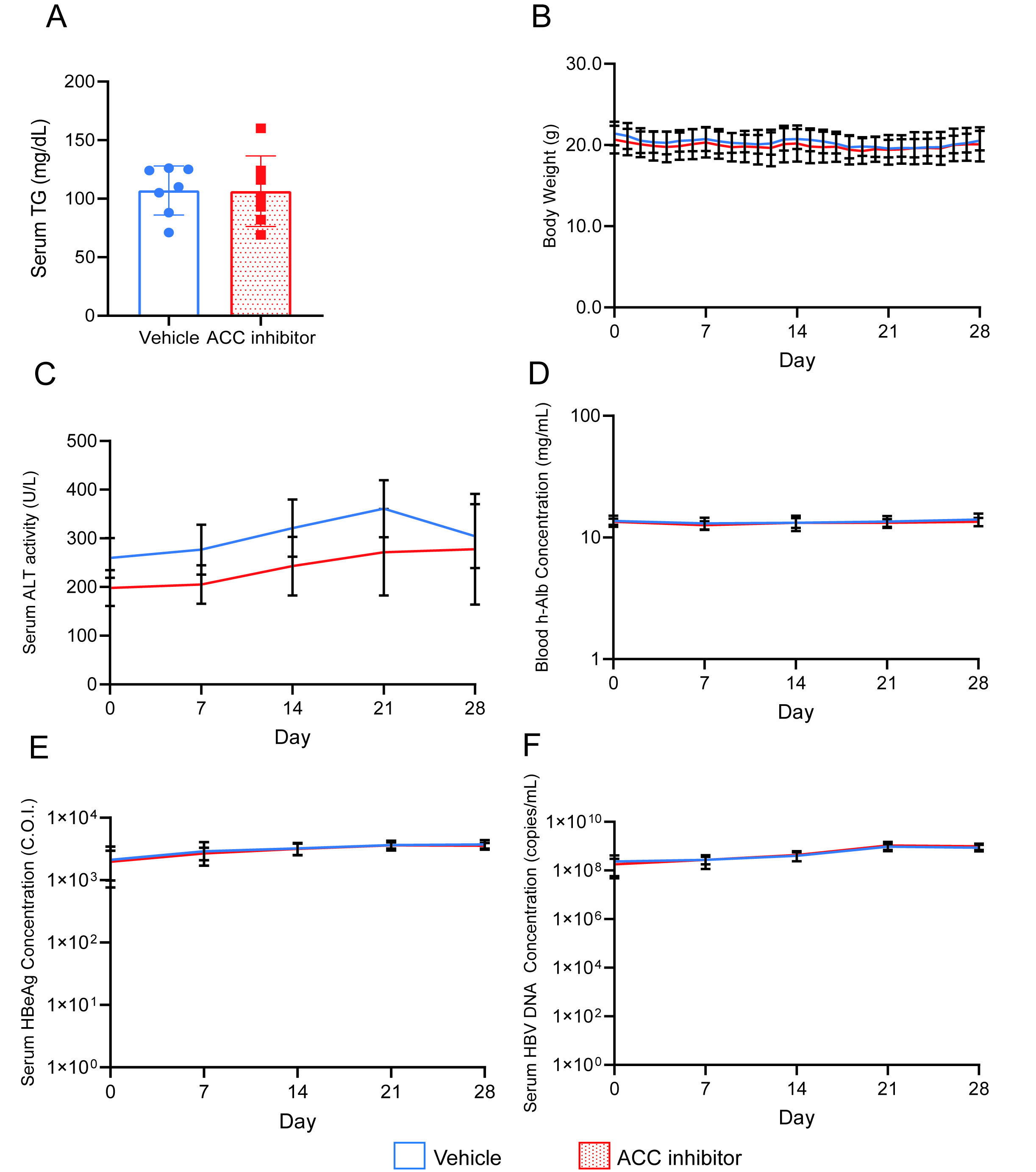

Supplement: S2 Fig — (A-F) Laboratory parameters measured during the study in ACC inhibitor- and vehicle-treated groups. Serum triglycerides levels 7 days before treatment (A); body weights (B); serum ALT activity (C); h-Alb concentration (D); serum HBeAg concentration (E); serum HBV DNA (F). Data is shown as mean +/- SD. (TIF) [file pone.0270273.s002.tif]
