# Supplementary material for: Targeting lipid biosynthesis pathways for hepatitis B virus cure
Source: PLoS One. 2022 Aug 4;17(8):e0270273. doi: 10.1371/journal.pone.0270273 (PMC9352027; doi:10.1371/journal.pone.0270273)
Supplement: S2 Table — (PDF) [file pone.0270273.s004.pdf]

S2 Table. Top dysregulated genes in the HBV-infected livers from chimeric humanized mice compared to non-infected mice based on RNAseq analysis.

Gene	logFC	AveExpr	P.Value	adj.P.Val
MDM2	0.439643	5.729676	1.1E-05	0.090299
RRS1	-0.90088	1.597069	2.87E-05	0.090299
DNAJB9	-0.30468	5.539102	2.93E-05	0.090299
DNAJA1	-0.41704	7.141932	3.65E-05	0.090299
GCH1	-0.44939	4.753297	3.92E-05	0.090299
GRAMD2B	0.60087	3.401326	5.12E-05	0.091752
ZFPM1	-0.5981	4.029819	5.58E-05	0.091752
JMJD1C	0.329415	5.743186	0.000118	0.144507
RICTOR	0.328365	4.690555	0.000125	0.144507
NAV2	-0.43841	5.191206	0.000127	0.144507
