# Supplementary material for: Targeting lipid biosynthesis pathways for hepatitis B virus cure
Source: PLoS One. 2022 Aug 4;17(8):e0270273. doi: 10.1371/journal.pone.0270273 (PMC9352027; doi:10.1371/journal.pone.0270273)
Supplement: S3 Table — (PDF) [file pone.0270273.s005.pdf]

S3 Table. Anti-HBV activity and toxicity of compounds modulating lipid pathways.

Common Name and Target	IUPAC	Activity in HBV-infected HepG2-NTCP cells		
		HBsAg, EC_{50} (nM)	HBeAg, EC_{50} (nM)	Cell viability, CC_{50} (nM)
ACC inhibitor	2-(1-((R)-2-(((1s,4S)-4-hydroxycyclohexyl)oxy)-2-(2-methoxyphenyl)ethyl)-5-methyl-6-(oxazol-2-yl)-2,4-dioxo-1,4-dihydrothieno[2,3-d]pyrimidin-3(2H)-yl)-2-methylpropanoic acid	29	9600	>10000
FASN inhibitor	4-(1-(5-(2-cyclopropyl-4-methyl-1H-imidazol-5-yl)-2,4-dimethylbenzoyl)-3-fluoroazetidin-3-yl)benzonitrile	81	>10000	>10000
SKI-1/S1P inhibitor	(R)-4-((diethylamino)methyl)-N-(2-methoxyphenethyl)-N-(pyrrolidin-3-yl)benzamide	790	>10000	>10000
JTE-013 S1P allosteric antagonist	N-(2,6-dichloropyridin-4-yl)-2-(4-isopropyl-1,3-dimethyl-1H-pyrazolo[3,4-b]pyridin-6-yl)hydrazine-1-carboxamide	6700	>10000	>10000
SKI-II Dual Sphingosine kinase inhibitor	4-((4-(4-chlorophenyl)thiazol-2-yl)amino)phenol	8836	>10000	>10000
CPT2 Inhibitor	2-((4-chloro-3-nitrophenyl)sulfonamido)benzoic acid	>10000	>10000	>10000
Etomoxir CPT1: Transport Inhibitor	ethyl (R)-2-(6-(4-chlorophenoxy)hexyl)oxirane-2-carboxylate	>10000	>10000	>10000
ACSS2 Inhibitor	N-(3-(1,1-difluoroethyl)phenyl)-1-(4-(difluoromethoxy)-3-(pyridin-3-yl)phenyl)-3-methyl-5-oxo-4,5-dihydro-1H-pyrazole-4-carboxamide	>10000	>10000	>10000
DGAT2 Inhibitor	3-(2-chlorophenyl)-1-(4-fluorophenyl)-N-(3-methyl-1,1-dioxidotetrahydrothiophen-3-yl)-1H-indole-6-carboxamide	>10000	>10000	>10000
Pradagilast, LCQ908 DGAT1: Inhibitor	2-((1r,4r)-4-(4-(5-((6-(trifluoromethyl)pyridin-3-yl)amino)pyridin-2-yl)phenyl)cyclohexyl)acetic acid	>10000	>10000	>10000
DGAT1 Inhibitor	2-((1r,4r)-4-(4-(4-amino-5-oxo-7,8-dihydropyrimido[5,4-f][1,4]oxazepin-6(5H)-yl)phenyl)cyclohexyl)acetic acid	>10000	>10000	>10000
ACLY Inhibitor	methyl 3-chloro-5-(N-(4,6-difluoro-[1,1'-biphenyl]-3-yl)sulfamoyl)-4-hydroxybenzoate	>10000	>10000	>10000
LXR Inverse Agonist	ethyl 5-(((2,4,6-trimethyl-N-((3'-(methylsulfonyl)-[1,1'-biphenyl]-4-yl)methyl)phenyl)sulfonamido)methyl)furan-2-carboxylate	>10000	>10000	>10000
KHK Inhibitor	2-((1R,5S,6R)-3-(2-((S)-2-methylazetidin-1-yl)-6-(trifluoromethyl)pyrimidin-4-yl)-3-azabicyclo[3.1.0]hexan-6-yl)acetic acid	>10000	>10000	>10000
SCD1 Inhibitor	(R)-4-((3S,5S,7R,8R,9S,10S,12S,13R,14S,17R)-7,12-dihydroxy-3-icosanamido-10,13-	>10000	>10000	>10000

	dimethylhexadecahydro-1H-cyclopenta[a]phenanthren-17-yl)pentanoic acid			
Fatostatin SREBP Processivity Blocker	2-(2-propylpyridin-4-yl)-4-(p-tolyl)thiazole	>10000	>10000	>10000
Betulin SREBP Processivity Blocker	(1R,3aS,5aR,5bR,7aR,9S,11aR,11bR,13aR,13bR)-3a-(hydroxymethyl)-5a,5b,8,8,11a-pentamethyl-1-(prop-1-en-2-yl)icosahydro-1H-cyclopenta[a]chrysen-9-ol	>10000	>10000	>10000
BioE-115 PASK (kinase) modulator	2-(4-fluorophenyl)-3-(isopropyl(methyl)amino)quinoxaline-6-carboxylic acid	>10000	>10000	>10000
FTY720 (S)-phosphate) S1PR functional antagonist	(S)-2-amino-2-(hydroxymethyl)-4-(4-octylphenyl)butyl dihydrogen phosphate	>10000	>10000	>10000
FTY720, Fingolimod TY 52156 S1PR functional antagonist	2-amino-2-(4-octylphenethyl)propane-1,3-diol	>10000	>10000	>10000
