# Supplementary material for: Targeting lipid biosynthesis pathways for hepatitis B virus cure
Source: PLoS One. 2022 Aug 4;17(8):e0270273. doi: 10.1371/journal.pone.0270273 (PMC9352027; doi:10.1371/journal.pone.0270273)
Supplement: S1 File — (PDF) [file pone.0270273.s006.pdf]

Supporting Materials and Methods

Quantification of Extracellular DNA by qPCR

Viral DNA from PHH supernatants was purified using the Qiagen DNeasy 96 kit (69582) following the manufacturer's recommended protocol. Quantification of vDNA by qPCR amplification of the HBx region of the genome was performed by combining 5 µL of DNA, 900 nM of HBx forward primer (GGACCCCTGCTCGTGTTACA), 900 nM of reverse primer (GAGAGAAGTCCACCACGAGTCTAGA) (Supplemental Table 4), 200 nM TaqMan probe ([6FAM] TGTTGACAAGAATCCTCACCAATACCAC [NFQ-MGB]), and 1x TaqMan Fast Advanced Master Mix (Thermo Fisher Scientific; 4444557) for a total reaction volume of 20 µL in 96-well PCR plates (Thermo Fisher Scientific; 4346906). qPCR was carried out on a real-time PCR system (Thermo Fisher Scientific; QuantStudio 7 Flex) using the following conditions: 95°C for 20 seconds, followed by 40 cycles of 95°C for 1 second and 60°C for 20 seconds. A plasmid containing the HBV full genome was used for the standard curve.

Quantification of Intracellular Viral RNA

Intracellular HBV viral RNA (vRNA) was isolated from PHH using the RNeasy 96 kit (Qiagen, 74182) following the manufacturer's recommended protocol. After elution, DNase digestion by Turbo DNase (Thermo Fisher Scientific; AM2239) was performed to remove any contaminating DNA. After 30 minutes of DNase treatment at 37°C, Turbo DNase was inactivated by adding 15 mM of EDTA (Fluka; 03690) and heating the reaction to 75°C for 10 minutes in a thermo cycler (Thermo Fisher Scientific; Veriti 96 Well Thermal Cycler). Quantification of vRNA by qRT-PCR (quantitative reverse transcription polymerase chain reaction) amplification of the HBx region of the genome was performed by combining 5 µL of RNA to 900 nM of HBx forward primer, 900 nM of HBx reverse primer, 200 nM TaqMan probe, and 1x beta-actin (ACTB) endogenous transcripts (Thermo Fisher Scientific; 4310881E) and 1x TaqMan Fast Virus 1-Step Master Mix

(Thermo Fisher Scientific; 4444434) for a total reaction volume of 20 µL in 96-well PCR plates (Thermo Fisher Scientific; 4346906). qRT-PCR was carried out on a real-time PCR system (Thermo Fisher Scientific; QuantStudio 7 Flex) using the following conditions: 50°C for 5 minutes, then 95°C for 20 seconds, followed by 40 cycles of 95°C for 3 seconds and 60°C for 30 seconds. ACTB mRNA expression was used to normalize target gene expression. Levels of HBV mRNA for all donors were calculated as fold change relative to no drug treated sample using the 2-ΔΔCt method.

Intracellular HBsAg by CLIA

Cell were lysed using M-PER Mammalian Protein Extraction Reagent, 1X (Thermo Fisher Scientific, #78501) with 1X Halt Protease and Phosphatase inhibitor (Thermo Fisher Scientific, #78443) for 10 min at room temperature. HBsAg levels were measured using HBsAg Chemiluminescence Immunoassay Kit (Autobio, CL0310-2) following the manufacturer's recommended protocol. Briefly, 50µl of HBsAg reference standards, specimens, and controls were dispensed into antibody coated microtiter plate with 50µl of Enzyme Conjugate Reagent. The samples were thoroughly mixed and incubated at 37 ℃ for 60 minutes. Following incubation, the mixture was discarded, and the microtiter plate was rinsed with washing buffer 6 times. 25µl of Substrate A and 25µl of Substrate B were added and incubated for 10 min before Related Light Unit (RLU) readout by SpectraMax M5 microplate reader (Molecular Devices).

Fluorescent microscopy

PHH were fixed in 4% paraformaldehyde and permeabilized with 0.3% Triton-X100 for 30 minutes at room temperature. Cells were stained with rabbit monoclonal anti-HBV core antibody (clone 366-2, final 1µg/ml, Gilead Sciences) and murine anti-HBsAg monoclonal antibody (XTL-17, final 1µg/ml, Gilead Sciences) diluted in PBS containing 0.3% Triton-X100 and 3% bovine serum albumin (BSA). After washing with PBS, bound antibodies were detected using Alexa Fluor®-555-conjugated goat anti-rabbit secondary antibody and Alexa Fluor®-488-conjugated

goat anti-mouse secondary antibody (Life Technologies) diluted 1:500 in PBS containing 0.3% Triton-X100 and 3% BSA. Nuclei were stained with 4’, 6-diamidine-2-phenylindole (DAPI) (Life Technologies). Images were taken with a confocal Opera Phenix high-content screening system equipped with a 20x objective lens. All images within each sample set were captured using identical confocal settings.
